# Supplementary material for: CALMS: Modelling the long-term health and economic impact of Covid-19 using agent-based simulation
Source: PLoS One. 2022 Aug 29;17(8):e0272664. doi: 10.1371/journal.pone.0272664 (PMC9423607; doi:10.1371/journal.pone.0272664)
Supplement: S1 File — Data and results of the validation and case study experiments conducted with the CoronAvirus Lifelong Modelling and Simulation (CALMS) model are available at https://doi.org/10.17633/rd.brunel.19350518. The repository also includes the R code used to generate the output graphs. (PDF) [file pone.0272664.s001.pdf]

Data and results of the validation and case study experiments conducted with the CoronAvirus Lifelong Modelling and Simulation (CALMS) model are available at https://doi.org/10.17633/rd.brunel.19350518.

The repository also includes the R code used to generate the output graphs.
