# Supplementary material for: CALMS: Modelling the long-term health and economic impact of Covid-19 using agent-based simulation
Source: PLoS One. 2022 Aug 29;17(8):e0272664. doi: 10.1371/journal.pone.0272664 (PMC9423607; doi:10.1371/journal.pone.0272664)
Supplement: S2 File — The CoronAvirus Lifelong Modelling and Simulation (CALMS) code is available on https://gitlab.com/anabrunel/calms. (PDF) [file pone.0272664.s002.pdf]

The CoronAvirus Lifelong Modelling and Simulation (CALMS) code is available on https://gitlab.com/anabrunel/calms.
