# Supplementary material for: CALMS: Modelling the long-term health and economic impact of Covid-19 using agent-based simulation
Source: PLoS One. 2022 Aug 29;17(8):e0272664. doi: 10.1371/journal.pone.0272664 (PMC9423607; doi:10.1371/journal.pone.0272664)
Supplement: S3 File — The file includes the CALMS Overview, Design concepts and Details (ODD) protocol. (PDF) [file pone.0272664.s003.pdf]

CALMS Overview, Design concepts and Details (ODD) protocol

CALMS: Modelling the long-term health and economic impact of Covid-19 using agent-based simulation.

Kate S. Mintram, Anastasia Anagnostou, Nana Anokye, Edward Okine, Derek Groen, Arindam Saha, Nura Abubakar, Tasin Islam, Habiba Daroge, Maziar Ghorbani, Yani Xue, Simon J.E. Taylor

Full model description

- *Overview*
  - *Purpose*
  - *Entities, state variables, and scales*
  - *Process overview and scheduling*
- *Design concepts*
  - *Basic Principles*
  - *Emergence*
  - *Adaptation*
  - *Interaction*
  - *Stochasticity*
  - *Observation*
- *Details*
  - *Initialisation*
  - *Input data*
  - *Sub-models*

Overview

Purpose:

The model was developed to predict the long-term health and economic impact of Covid-19, and associated preventative and/or therapeutic interventions, on a population or a subgroup of a population. CALMS considers intervention uptake levels and tracks the characteristics of individuals over the simulation, such that disease outcomes are a function of each individual's health status (e.g. age, sex, comorbidities).

Entities, state variables and scales:

The entities in the model are the spatial units (landscape grid) and individual people (agents).

The model is initialised so that each spatial unit on the grid contains one agent. At initialisation, each agent in the model is characterised by their age, sex, ethnicity, physical activity status, body mass index (BMI), blood pressure, high-density lipoprotein (HDL) ratio, education, deprivation, socio-economic class as well as whether they have cardiovascular disease (CVD) or a family history of CVD, diabetes or a family history of type 2 diabetes (T2D), blood pressure treatment, rheumatoid arthritis, chronic kidney disease, depression, musculoskeletal injuries (MSI), and smoking history. As the model runs, age, BMI, HDL ratio, and blood pressure are updated, along with physical activity status, CVD and T2D, depression and MSI.

Agents are additionally characterised by whether they are susceptible, exposed, infected or have recovered from Covid-19. They also have a vaccination status and a lockdown eligibility status. Infected agents may demonstrate mild, severe or critical disease outcomes, and be admitted to hospital or ICU accordingly for a given length of stay (LOS). Exposed or infected agents have a given time period that they are exposed or infected for. Recovered individuals may develop Long Covid. All agents are characterised as being dead or alive, and dead agents have an associated cause of death.

Agents have costs associated with a given health event (MSI, depression, CVD, diabetes), and costs associated with Covid-19 hospital/ICU admissions as well vaccinations and lockdowns.

The temporal scales of the model reflect the lifetime of the cohort. The user can set an end time (within one-year increments) or by default the model runs until all agents are dead. Whilst the model is not spatially explicit, the user interface makes use of a grid so that status of the agents can be visually tracked.

Process overview and scheduling:

Each of the following processes (in bold) will occur over each time step in sequential order. Entities are processed in a random sequence and individual's update their state variables each day with the exception of age, non Covid-19 related mortality, and risks of developing CHD, diabetes, stroke, depression and MSI which are updated every 3 months. Fig A1 shows the conceptualisation of the process overview and scheduling.

Calculate risk of developing T2D, stroke and CHD: These non-communicable diseases (NCDs) carry a mortality risk and contribute to the risk of developing severe or critical Covid-19 outcomes. Within this sub-model, the risk of developing MSI and depression are additionally updated and these NCDs carry a mortality risk.

Update baseline Physical Activity status: The trajectory of lifelong physical activity is a function of previous activity levels as well as the characteristics of the agent. The risks of developing all NCDs (described above) are a function the physical activity status of the individual.

Update lockdown scenario and calculate costs: Agents who meet the age criteria for a lockdown reduce the number of contacts they interact with per day accordingly. Each eligible agent is designated a daily cost associated with the lockdown.

Calculate infection probability: All susceptible agents in the model determine if they are exposed to Covid-19 each day via a dynamic infection probability based on the number of infected individuals in the cohort, the transmission probability of the virus and the number of contacts they encounter each day. After a set exposure period, exposed agents become infected.

Update vaccination scenario and calculate costs: Agents who meet the specified criteria (age, heath status) and have chosen to uptake vaccination adjust their risk of developing severe or critical Covid-19 accordingly. Each eligible agent is designated a cost associated with each vaccination they receive. Boosters are administered to agents who chose to have the initial vaccination every 6 months.

Calculate risks and update severity: On the day that agents become infected with Covid-19, they calculate their risk of developing severe or critical disease and thus their risk of being admitted to hospital or ICU, respectively. The risk probability is a function of the agent's characteristics and comorbidities.

Update hospital/ICU duration and cost: Agents with severe or critical disease calculate a hospital or ICU length of stay, respectively, according to a gamma distribution. A designated daily cost is assigned to each agent.

Check Covid-19 related death and long-term effects: Agents in hospital or ICU die according to a given daily death probability. Agents with mild, severe or critical disease can develop long-term health effects (Long Covid) according to a given probability.

Check recovery and update variables: If an agent is discharged from hospital or ICU or displayed mild symptoms and is no longer infectious, they are recovered and all disease related variables are reset.

Update age and check death from other causes: All living agents in the simulation age and check if death has occurred from non Covid-19 related causes, including depression, MSI, CHD, diabetes, stroke, age, and other background mortalities. Baseline mortality according to life tables for the age and sex groups is also considered. This step occurs every 3 months.

Record total economic costs: Total healthcare costs (hospital and ICU) and intervention costs (vaccination and lockdown) for the cohort are summarised. Costs of NCDs are additionally calculated.

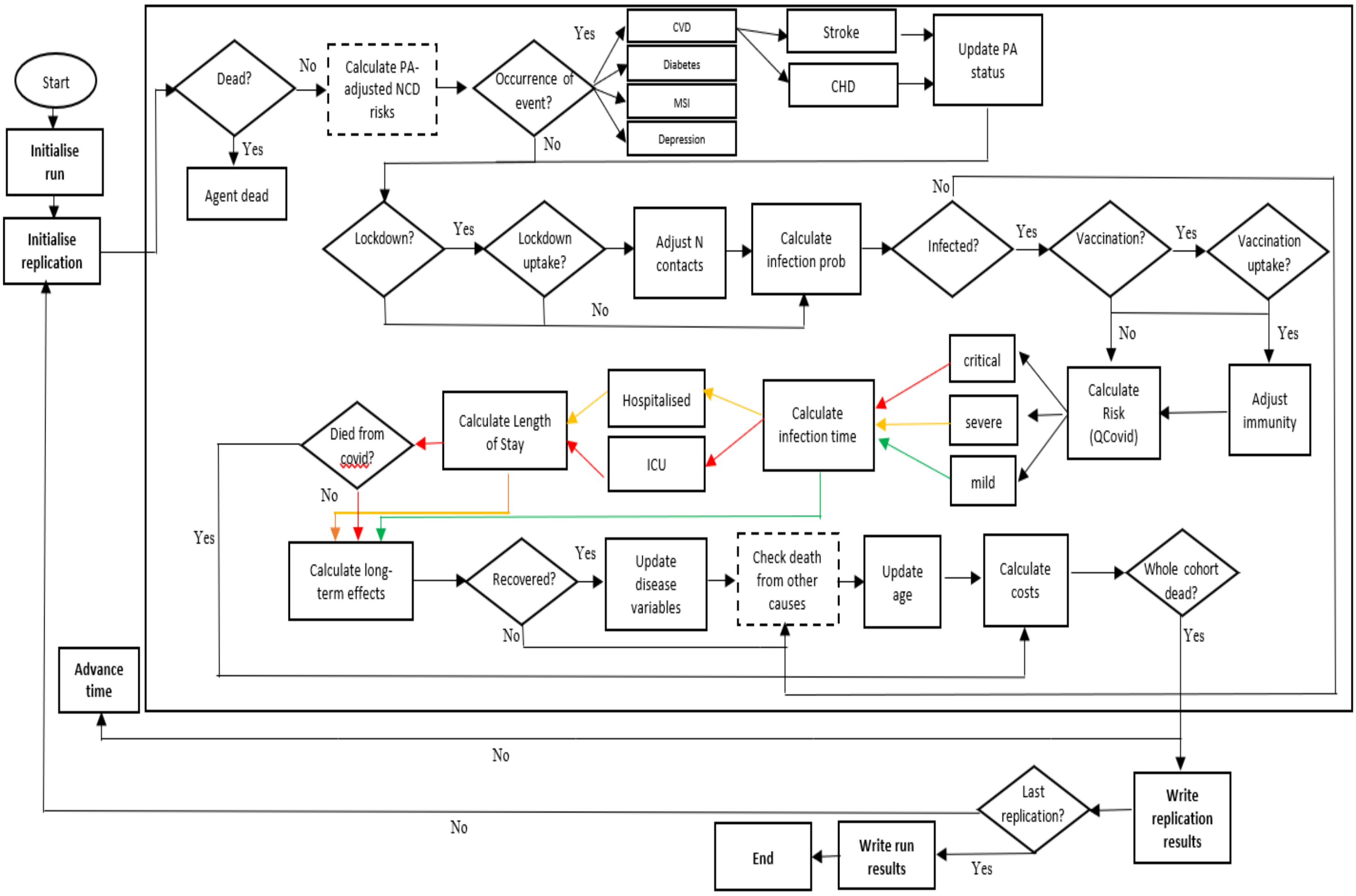

Figure A1. Conceptualisation of the CALMS model. Each agent undertakes the processes within the weighted black box each time step, with the exception of the dashed boxes which are undertaken every three months. PA=physical activity; NCD=non-communicable disease; CVD=cardiovascular disease; MSI=musculoskeletal injuries.

Design concepts

Basic Principles: CALMS follows the basic principles of a 'Susceptible, Exposed, Infected, Recovered' (SEIR) model, whereby susceptible agents may become exposed, and subsequently infected with Covid-19 according to an infection probability. At the end of the infected period, individuals either die, recover, or develop long-term effects. The model uses established risk algorithms (QDiabetes, QRisk, QCovid) to determine risks of developing NCDs and disease outcomes of Covid-19.

Emergence: Covid-19 outcomes emerge from the characteristics (e.g. age, BMI) and health status (Covid-19 comorbidities) of the agents. The probability of an NCD event occurring also emerges from the characteristics and health status of the agents, as well as their physical activity levels. In turn, the number of hospitalisations, ICU admissions and Covid-19 related fatalities, along with associated healthcare costs, are emergent properties of the model.

Adaptation: The key adaptive behaviour of agents in the model is the decision to uptake, or reject, a vaccination intervention based on a given uptake probability. The objective of accepting a vaccination is to reduce the risk of severe or critical disease outcomes.

Interaction: Only indirect interaction occurs in the model. Agents have a set number of contacts with whom they are exposed to each day, and this is adapted if there is a lockdown in place. These contacts may or may not be infected with Covid-19, based on the number of infected agents within the population. The number of contacts an agent encounters which are infected determines the infection probability of that agent.

Stochasticity: All of the probabilities in the model are generated using random numbers. This includes infection probabilities, risk of developing severe or critical Covid-19 disease, risk of developing NCDs, the probability of developing Long Covid, and death probabilities. In addition, the time period between initial infection and re-infection, the length of stay in hospital/ICU, the exposure period of the virus, and the duration of Long Covid are drawn from statistical distributions.

Observation: The user interface displays daily updates of infection rates, hospital and ICU admissions and fatalities, as well tallying the total healthcare and intervention costs. In addition, at the end of each simulation replication, individual life histories for all agents are generated, including all events that occurred on a specific agent in their lifetime. This feature requires to keep a memory of all events per individual and therefore is computationally expensive. For this reason, it is optional and can be selected only if the objectives of the simulation study require it.

At cohort level, for each simulation replication annual and end of simulation averages are recorded. At the end of the simulation run, cohort averages for all replications are recorded. Outputs include NCD events, number of infections, hospital/ICU admissions, Covid-19

fatalities, Long Covid cases, as well as healthcare and intervention costs. By default, all population averages (annual, end of replication and end of run) are exported to CSV files.

Details

Initialisation:

Population initialisation: The model includes a sample dataset of individuals drawn from health surveys. Each record represents an individual in the population and its characteristics. Population characteristics include demographics as well as socio-economic and health characteristics such as age, sex, ethnicity, education, deprivation, body mass index (BMI), systolic blood pressure (hypertension), type 1 and type 2 diabetes, family history of cardiovascular disease (CVD), stroke, diabetes, kidney disease, etc. Currently, the CALMS population dataset includes 9,594 records that reflect the English population over five years of age based on the 2012 Health Survey for England [1]. A cohort comprised of population subgroups can be generated. The selection is based on the individual characteristics i.e. by age, sex, medical history, etc. and can include one or a combination of these characteristics. Subgroups can be selected both as a population base and as a target population group for an intervention. The intervention target population subgroup can be the whole population base or a subset of it. The population cohort size can also be selected at initialisation. If the selected cohort size is greater than the population dataset, a synthetic population is generated using sampling with replacement to inflate the cohort size based on the seed population.

Table 1 shows a summary of the sample population dataset.

Table 1. Selected baseline characteristics of the initial population (n = 9,594)

Female, n (%)	5,268 (54.9)
Age, (years) median (25th, 75th percentile)	45 (27, 63)
Ethnicity, n (%)	
White	8,470 (88.3)
Black	247 (2.5)
Asian	642 (6.6)
Other	235 (2.4)
BMI, median (25th, 75th percentile)	25.95 (22.21, 29.72)
CVD history, n (%)	1,033 (10.8)
Type 2 Diabetes, n (%)	527 (5.5)

Model initialisation: The model begins on 31st January, when the first case of Covid-19 were modelled in the UK. The number of susceptible and exposed individuals within the population is user set, but is set to a default of 100% and 10 individuals, respectively. The environment is initialised as a grid, the size of which depends on the number of agents within the cohort, with each agent occupying a single grid cell.

Input data:

Table 2. CALMS input parameter names and values, sources and additional information.

Parameter	Value	Additional information	Reference
Transmission Probability	0.022	Calculated using an R0 value of 2.2-2.7 and N contacts 10.8.	[2,3]
N Contacts (no lockdown)	10.8		[4]
N Contacts (lockdown)	2.8		[4]
Exposure Period (days - mode (min, max))	3.5 (0,12)	Calculated in the model using a triangular distribution.	[5]
Infectious Period (days)	9	Considers a pre-symptomatic infectious period of 2 days.	[6,7]
Time from infectious to hospitalisation (days)	9	Considers a pre-symptomatic infectious period of 2 days.	[8]
Hospital/ ICU LOS (days - mean ± sd)	12.8 (13.4) / 13.2(13.4)		[9]
ICU Probability	0.17		[10]
Death Probability (ICU)	0.32		[10]
Death Probability (Hospital)	0.26		[10]
Long Covid Probability	0.033	Clinical data based on reported infections. Parameter value considers that the CDC estimate number of actual cases to be 4.3 times reported cases.	[11, 12]

Time to reinfection (months)	6 - 12	Data suggests possible reinfection after 6 months, modelled as a range between 6 and 12 months for stochasticity.	[13]
Duration of long covid (weeks)	4 - 77	The maximum duration of long-covid is currently unclear, data suggests at least a year.	[14]
Vaccine immunity	0.93		[15,16,17]
Vaccine Uptake	0.96		[18]
Hospital/ ICU cost (£)	797/£1681		[19]
Vaccine cost per capita (£)	1.57	Cost only includes actual cost of the vaccine dose (no R&D, administration etc).	[20]
Lockdown cost per capita (£)	17.53	Calculated using the fall in GDP between Apr – June 2020 during lockdown restrictions (19.5%).	[21]
QCovid multiplication factor	9		*

* The multiplication factor has been derived from calibrating the model so we calculate only the risk for hospitalisation using the QCovid risk algorithm. The method is explained in the experimental design section of the main paper.

Sub-models:

Calculate risks of developing T2D, stroke and CHD: The risk of developing CVD and T2D are calculated according to the established QRisk2 [22] and QDiabetes [23] algorithms respectively. The risk of coronary heart disease (CHD) or a stroke episode is a probability of a CVD event. The NCD risks are calculated every three months. These risks are then adjusted considering the physical activity status of the individual. All risks are a function of the agent's demographic, socio-economic and medical characteristics.

This sub-model additionally calculates the physical activity adjusted risk of developing depression and MSI, as described in Anagnostou et al [24]. Both of these NCDs contribute to an agent's death probability but are not considered comorbidities of Covid-19 in the model.

Update Physical Activity Status: Each agent in the model has a baseline physical activity status. Physical activity levels are classified into three categories using minutes of vigorous physical activity (MVPA) per week as a measure: Inactive (0 < physical activity status < 85 min per week); Moderately active (86 < physical activity status < 425 min per week); and Very active (physical activity status). Individuals can switch activity categories during their lifetime. The formulae for calculating physical activity changes over time are shown in Anagnostou et al. [24]. The trajectory of lifelong physical activity is a function of previous activity levels as well as the characteristics of the agent. The risks for CVD and T2D are adapted in CALMS to incorporate the relative risk for developing a condition based on the physical activity level of individuals.

Update lockdown scenario and calculate costs: A lockdown can be introduced if the global conditions (start date, end date) and agent characteristics (age) meet the criteria set at initialisation. A lockdown carries a specific cost and aims to reduce the number of exposures an agent encounters, considering their likelihood of adhering to the lockdown, which subsequently reduces their infection probability. The number of contacts individuals come into contact with during a lockdown was quantified from the CoMix survey undertaken by Jarvis et al. [4].

Calculate infection probability: All agents are susceptible to Covid-19 infection at initialisation. An agent's infection probability is determined by the probability of infection per exposure (i.e., transmission probability [2,3]) and the number of exposures per time step [4] according to the equation Eq (1):

$$I_p = T_p C_n C_i$$

Where I_p denotes infection probability, T_p denotes the transmission probability of the virus, C_n denotes the number of contacts each agent comes into contact with each day, and C_I denotes the proportion of agents in the cohort which are infectious. If the infection probability is greater than a random number drawn between 0 and 1, an agent becomes infected and is no longer susceptible. The exposure (i.e., pre-infectious) period of the virus for each infected agent is then calculated from a triangular distribution, using data from WHO, [5]. If an agent is not infected, the following disease related steps are not implemented and they will proceed to updating their age, checking death from other causes and recording their total costs. An agent becomes susceptible to Covid-19 infection again 6-12 months after the previous infection [13].

Update vaccination scenario and calculate costs: A vaccination programme can be introduced if the global conditions (start date, end date) and agent characteristics (age, health status) meet the criteria set at initialisation. A vaccine carries a specific economic cost [20] and aims to reduce the risk of agents developing severe or critical symptoms. Agents decide if they are going to uptake the vaccine, according to a given uptake probability [18], and their risk is adjusted accordingly. The proportion of the cohort that are vaccinated per day is specified as an input parameter and determines the speed of the vaccination programme. Vaccinated agents acquire full immunity following a given time period of 54 days to account for the time between

the two vaccine doses (calculated as the mean manufacturer recommended duration between doses of Phizer (21 days [25]), Moderna (28 days [25]) and AstraZeneca (8-12 weeks [26])) as well as the 14-day time lag between the administration of the second dose and obtaining full immunity. Vaccination immunity is assumed to last the same amount of time as natural immunity. Booster vaccinations are administered every 6 months; however, this time period can be set as an input parameter.

Calculate risks and update severity: The risk of developing mild or severe symptoms is calculated given an agent's characteristics (e.g. age, sex, ethnicity, bmi) and comorbidities (e.g. spb, CHD, T2D, T1D, stroke, kidney disease) according to the QCovid risk estimation algorithm [27]. If an agent has severe symptoms, their risk of developing critical symptoms is calculated according to a given probability of being admitted to ICU [10]. Vaccine immunity alters this risk probability accordingly (set to 0 if there is no vaccination programme in place or the conditions for vaccine uptake are not met). Agents infected with mild coronavirus have a set infectious period of 9 days [6,7]; severe or critical cases are infectious for the duration of their time in hospital/ICU.

Update hospital/ICU duration and cost: Agents with severe or critical disease are admitted to hospital or ICU, respectively, following a set time period of 9 days between becoming infected with the virus and hospitalisation [8]. The length of stay is calculated from a gamma distribution using data from Baillie et al. [9] and an associated cost of stay is calculated [19].

Check Covid-19 related death and long-term effects: Agents in hospital or ICU die according to a given daily death probability [10]. Agents with mild, severe or critical disease can develop long-term health effects (Long Covid) according to a given probability [11,12]. All infected agents have the same probability of developing Long Covid, and Long Covid does not currently affect risks for developing T2D or CVD.

Check recovery and update variables: If an agent is discharged from hospital or ICU or displayed mild symptoms and is no longer infectious, they are recovered and all disease related variables are reset.

Update age and check death from other causes: All living agents in the simulation age and check if death has occurred from non Covid-19 related causes, including depression, musculoskeletal injuries (MSI), CHD, diabetes and stroke. Baseline mortality according to life tables for the age and sex groups is also considered, as described in Anagnostou et al. [24]. This step occurs every 3 months.

Record total economic costs: Total healthcare costs (hospital and ICU [19]) and intervention costs (vaccination [20] and lockdown [21] for the cohort are summarised. Costs of NCDs are additionally calculated as described in Anagnostou et al. [24].

<u>References</u>*:*

[1] HSE Health Survey for England. https://digital.nhs.uk/data-and-information/publications/statistical/healthsurvey-for-england/health-survey-for-england-2012. (Accessed 6 March 2022). 2013.

[2] Li Q, Guan X, Wu P, Wang X, Zhou L, Tong Y, Ren R, Leung KS, Lau EH, Wong JY, Xing X. Early transmission dynamics in Wuhan, China, of novel coronavirus‑infected pneumonia. New England journal of medicine. 2020 Jan 29. 24.

[3] Wu J, Tang B, Bragazzi NL, Nah K, McCarthy Z. Quantifying the role of social distancing, personal protection and case detection in mitigating COVID-19 outbreak in Ontario, Canada. Journal of mathematics in industry. 2020 Dec;10(1):1-2.

[4] Jarvis CI, Van Zandvoort K, Gimma A, Prem K, Klepac P, Rubin GJ, Edmunds WJ. Quantifying the impact of physical distance measures on the transmission of COVID-19 in the UK. BMC medicine. 2020 Dec;18(1):1-0.

[5] World Health Organization (WHO). Transmission of SARS-CoV-2: implications for infection prevention precautions: scientific brief, 09 July 2020. World Health Organization; 2020.

[6] Byrne AW, McEvoy D, Collins AB, Hunt K, Casey M, Barber A, Butler F, Griffin J, Lane EA, McAloon C, O' Brien K. Inferred duration of infectious period of SARS-CoV-2: rapid scoping review and analysis of available evidence for asymptomatic and symptomatic COVID-19 cases. BMJ open. 2020 Aug 1;10(8):e039856.

[7] Cevik M, Tate M, Lloyd O, Maraolo AE, Schafers J, Ho A. SARS-CoV-2, SARS-CoV, and MERS-CoV viral load dynamics, duration of viral shedding, and infectiousness: a systematic review and meta-analysis. The lancet microbe. 2021 Jan 1;2(1):e13-22.

[8] Faes C, Abrams S, Van Beckhoven D, Meyfroidt G, Vlieghe E, Hens N. Time between symptom onset, hospitalisation and recovery or death: statistical analysis of Belgian COVID-19 patients. International journal of environmental research and public health. 2020 Jan;17(20):7560.

[9] Baillie JK, Beane A, Blumberg L, Bozza F, Fowler RA, Barrio NG, Hashmi M, Jassat W, Laouenan C, Mentre F, Merson L. ISARIC COVID-19 Clinical Data Report: 8 April 2021. medRxiv. 2021 Jan 1:2020-07.

[10] Docherty AB, Harrison EM, Green CA, Hardwick HE, Pius R, Norman L, Holden KA, Read JM, Dondelinger F, Carson G, Merson L. Features of 20 133 UK patients in hospital with covid-19 using the ISARIC WHO Clinical Characterisation Protocol: prospective observational cohort study. bmj. 2020 May 22;369.

[11] Daugherty SE, Guo Y, Heath K, Dasmarinas MC, Jubilo KG, Samranvedhya J, Lipsitch M, Cohen K. SARS-CoV-2 infection and risk of clinical sequelae during the post-acute phase: A retrospective cohort study. medRxiv. 2021 Jan 1.

[12] Centre for Disease Control and Prevention (CDC). Estimated Covid-19 burden. https://www.cdc.gov/coronavirus/2019-ncov/cases-updates/burden.html. (Accessed 6 March 2022). 2021.

[13] Public Health England (PHE) Information on COVID-19 reinfection surveillance in England. https://www.gov.uk/government/publications/national-covid-19-reinfectionsurveillance/information-on-covid-19-reinfection-surveillance-in-england (Accessed 6 March 2022). 2021.

[14] New Scientist and Press Association Long covid has lasted over a year for 376,000 people in the UK. https://www.newscientist.com/article/2279878-long-covid-haslasted-over-a-year-for-376000-people-in-the-uk/ (Accessed 6 March 2022). 2021 Jun 4.

[15] Baden LR, El Sahly HM, Essink B, Kotloff K, Frey S, Novak R, Diemert D, Spector SA, Rouphael N, Creech CB, McGettigan J. Efficacy and safety of the mRNA-1273 SARS-CoV-2 vaccine. New England Journal of Medicine. 2020 Dec 30.

[16] Polack FP, Thomas SJ, Kitchin N, Absalon J, Gurtman A, Lockhart S, Perez JL, Marc GP, Moreira ED, Zerbini C, Bailey R. Safety and efficacy of the BNT162b2 mRNA Covid-19 vaccine. New England Journal of Medicine. 2020 Dec 10.

[17] Voysey M, Clemens SA, Madhi SA, Weckx LY, Folegatti PM, Aley PK, Angus B, Baillie VL, Barnabas SL, Bhorat QE, Bibi S. Safety and efficacy of the ChAdOx1 nCoV-19 vaccine (AZD1222) against SARS-CoV-2: an interim analysis of four randomised controlled trials in Brazil, South Africa, and the UK. The Lancet. 2021 Jan 9;397(10269):99-111.

[18] Office for National Statistics. Coronavirus and vaccine hesitancy, Great Britain Coronavirus and vaccine hesitancy, Great Britain: 9 August 2021. 2021. Available from: https://www.ons.gov.uk/peoplepopulationandcommunity/healthandsocialcare/healthandwellbeing/bulletins/coronavirusandvaccinehesitancygreatbritain/9august2021

[19] National Health Service (NHS). NHS offers COVID jab to clinically vulnerable and people 65 to 69. https://www.england.nhs.uk/2021/02/nhs-offers-covid-jabto-clinically-vulnerable-and-people-65-to-69/ (Accessed 6 March 2022). 2021

[20] Dyer O. Covid-19: countries are learning what others paid for vaccines. BMJ: British Medical Journal (Online). 2021 Jan 29;372.

[21] Office for National Statistics (ONS). Gross Domestic Product. https://www.ons.gov.uk/economy/grossdomesticproductgdp (Accessed 6 March 2022). 2020.

[22] Hippisley-Cox J, Coupland C, Vinogradova Y, Robson J, Minhas R, Sheikh A, Brindle P. Predicting cardiovascular risk in England and Wales: prospective derivation and validation of QRISK2. bmj. 2008 Jun 26;336(7659):1475-82.

[23] Hippisley-Cox J, Coupland C. Development and validation of QDiabetes-2018 risk prediction algorithm to estimate future risk of type 2 diabetes: cohort study. bmj. 2017 Nov 20;359.

[24] Anagnostou A, Taylor SJ, Groen D, Suleimenova D, Anokye N, Bruno R, Barbera R. Building global research capacity in public health: the case of a science gateway for physical activity lifelong modelling and simulation. In2019 Winter Simulation Conference (WSC) 2019 Dec 8 (pp. 1067-1078). IEEE.

[25] Kriss JL, Reynolds LE, Wang A, Stokley S, Cole MM, Harris LQ, Shaw LK, Black CL, Singleton JA, Fitter DL, Rose DA. COVID-19 vaccine second-dose completion and interval between first and second doses among vaccinated persons—United States, December 14, 2020− February 14, 2021. Morbidity and Mortality Weekly Report. 2021 Mar 19;70(11):389.

[26] AstraZeneca. COVID-19 Vaccine AstraZeneca receives interim recommendations for use by World Health Organization experts. https://www.astrazeneca.com/media-centre/articles/2021/covid-19-vaccine-astrazeneca-receives-interim-recommendations-for-use-by-world-health-organization-experts.html (Accessed 14.03.2022) 2021 Feb 10.

[27] Clift AK, Coupland CA, Keogh RH, Diaz-Ordaz K, Williamson E, Harrison EM, Hayward A, Hemingway H, Horby P, Mehta N, Benger J. Living risk prediction algorithm (QCOVID) for risk of hospital admission and mortality from coronavirus 19 in adults: national derivation and validation cohort study. bmj. 2020 Oct 20;371.
