# Supplementary material for: CALMS: Modelling the long-term health and economic impact of Covid-19 using agent-based simulation
Source: PLoS One. 2022 Aug 29;17(8):e0272664. doi: 10.1371/journal.pone.0272664 (PMC9423607; doi:10.1371/journal.pone.0272664)
Supplement: Attachment — Submitted filename: 1.ResponseToReviewers-v1.pdf [file pone.0272664.s005.pdf]

CALMS: Modelling the long-term health and economic impact of Covid-19 using agent-based simulation

Kate Mintram, Anastasia Anagnostou, Nana Anokye, Edward Okine, Derek Groen, Arindam Saha, Nura Abubakar, Tasin Islam, Habiba Daroge, Maziar Ghorbani, Yani Xue, and Simon J.E. Taylor

PONE-D-22-07757

We thank the Editors and Reviewers for the opportunity to revise our article. In particular we are very grateful for the additional time to revise this manuscript.

This document details our responses to reviewers.

===========================

Editor's Comments to Authors

===========================

Journal Requirements:

1. Please ensure that your manuscript meets PLOS ONE's style requirements, including those for file naming.

We thank the Editor. We believe the submitted files meet all of the PLOS ONE's requirements.

2. Thank you for stating the following in the Acknowledgments Section of your manuscript:

"This work was partially funded by the EU H2020 STAMINA project No. 883441."

We note that you have provided funding information. However, funding information should not appear in the Acknowledgments section or other areas of your manuscript. We will only publish funding information present in the Funding Statement section of the online submission form.

Please remove any funding-related text from the manuscript and let us know how you would like to update your Funding Statement. Currently, your Funding Statement reads as follows:

"KM, AA, NA, EO, DG, AS, NA, TI, HD, MG, YX and ST have received funding from the EU STAMINA project No. 883441 (https://cordis.europa.eu/project/id/883441).

The funders had no role in study design, data collection and analysis, decision to publish, or preparation of the manuscript."

Please include your amended statements within your cover letter; we will change the online submission form on your behalf.

We have removed the Acknowledgements section from the manuscript. We do not wish to amend the Funding Statement.

===========================

Reviewers' Comments to Authors

===========================

Referee #1

Review Comments to the Authors

This paper examines the lifelong health and economic impacts of Covid-19 and assesses the impact of related government interventions. The CoronAvirus Lifelong Modelling and Simulation (CALMS) model, an agent-based model, is used. The model is demonstrated by assessing the impact of multiple layered vaccination scenarios and/or lockdown measures on health and economic outcomes for the Covid 19 pandemic in the UK (UK or England please clarify). The results, i.e. the predictions for hospitalisations, ICU admissions and deaths, are plausible. This is a well-written paper whose strength is the use of the ABM model and the different scenarios. I have only a few comments.

Specific comments

The abstract is a bit long and only one third of the abstract deals with the results (which I find the most interesting). Report a bit more the results of the five scenarios.

We thank the referee for these comments. We have revised the abstract as follows. We have removed part of the details about the model and have added more information about the scenarios tested in our experimentation.

"The aim of this paper is to demonstrate our approach and present the long-term health and economic impact of Covid-19 in the UK."

Please add a sentence or two (data, method etc)

Addressed below.

At the end of the introduction, the authors state the aim of the paper, which is to present the long-term health and economic impact of Covid-19 in the UK using an agent-based model. Please add a sentence about the contribution of the paper to the literature. Is it the first study to use ABM in this field? Is the CALMS model new? Maybe this is the contribution (it is mentioned before):

This model takes into account the impact of individual health status on disease progression and also the long-term or lifelong health impact of Covid-19 on the population.

At the end of the introduction, we have added the following to make clear the data, methods and the contribution to the literature:

"The aim of this paper is to demonstrate our approach and present the long-term health and economic impact of Covid-19 in the UK. To the best of our knowledge, this is the first study that considers demographic, health, socio-economic and cost data and predicts lifelong risks of non-communicable diseases and the impact of Covid-19 and Long Covid based on comorbidities for the lifetime of a cohort."

"CALMS input parameter names and values, sources and additional information"

How sensitive are the results when the assumptions vary?. This is a crucial issue for all ABM applications.

We agree with the referee that sensitivity analysis is crucial in any stochastic model. CALMS has many input parameters that take a range of values and these individually or in combination could affect the results. We agree that assessing the robustness of the results is limited without this information. We would like however to keep the scope of this paper on presenting our model and its capabilities. We do not want to sound like we are avoiding this issue we would rather focus this paper on validation and demonstration of the capabilities of CALMS, i.e., studying targeted

interventions among others, and report on detailed sensitivity analysis in a subsequent publication. We hope that this is acceptable by the referee.

Check formula 1: No subscripts for i and t?

We would like clarification on this please. The elements of the equation are I_p, T_p C_n and C_I. We believe their meaning is explained but if the referee requests more information we are happy to provide it.

The discussion section contains all the elements, a brief summary of the results, implications, limitations and future work. There is little I could suggest for improvement.

This is a great study.

We would like to thank the referee for the constructive comments.

Referee #2

Review Comments to the Authors

Comments on "CALMS: Modelling the long-term health and economic impact of Covid-19 using agent-based simulation" (Manuscript Number: PONE-D-22-07757)

This paper develops a Covid-19 analysis system, which consider the long-term health and economic impact. The model is interesting but still has some problems that need to be explained, which are list as following:

1. Authors should explain how to set these parameters in Tab. 2

We thank the referee for his/her comment. To clarify this point, we have added the following description in the text under section "CALMS algorithm":

"All input parameters are set in a CSV file which is read at initialisation."

2. This model considers the economic impact of covid-19, but it only shows the impact of the disease on cost, how would the economic impacts the cure of disease?

This is a very interesting point and indeed it would be very useful to identify the impact of economics on the cure of the disease. However, this area of research is beyond the capabilities of our model and therefore we cannot comment on it. We hope that this is acceptable by the referee.

3. The model consider intervention. Such intervention would influence the spreading of disease, so is the transmission probability variable?

We have added the following sentence under "CALMS algorithm: calculate infection probability" section to clarify this point:

"The transmission probability T_p is constant for a specific virus variant. The infection probability depends on the infectious population and the number of contacts an individual has per time unit (day), as per Eq 1."

4. Please consider the following related papers

[1] Chuangxia Huang, Jie Cao, Fenghua Wen, Xiaoguang Yang. Stability analysis of SIR model with distributed delay on complex networks[J]. PLOS ONE, 2016, 11(8).

[2] Huang C, Zhang H, Cao J, et al. Stability and Hopf bifurcation of a delayed prey–predator model with disease in the predator[J]. International Journal of Bifurcation and Chaos, 2019, 29(07): 1950091.

[3] Jie Cao, Fenghua Wen, H E Stanley, Xiong Wang. Multilayer financial networks and systemic importance: Evidence from China[J]. International Review of Financial Analysis, 2021: 101882.

We have added the references, as suggested.
