# Supplementary material for: CALMS: Modelling the long-term health and economic impact of Covid-19 using agent-based simulation
Source: PLoS One. 2022 Aug 29;17(8):e0272664. doi: 10.1371/journal.pone.0272664 (PMC9423607; doi:10.1371/journal.pone.0272664)
Supplement: Attachment — Submitted filename: Comments on.docx [file pone.0272664.s004.docx]

Comments on “CALMS: Modelling the long-term health and economic impact of Covid-19 using agent-based simulation” (Manuscript Number: PONE-D-22-07757)

This paper develops a Covid-19 analysis system, which consider the long-term health and economic impact. The model is interesting but still has some problems that need to be explained, which are list as following:

1. Authors should explain how to set these parameters in Tab. 2
2. This model considers the economic impact of covid-19, but it only shows the impact of the disease on cost, how would the economic impacts the cure of disease?
3. The model consider intervention. Such intervention would influence the spreading of disease, so is the transmission probability variable?
4. Please consider the following related papers
[1] Chuangxia Huang, Jie Cao, Fenghua Wen, Xiaoguang Yang. Stability analysis of SIR model with distributed delay on complex networks[J]. *PLOS ONE*, 2016, 11(8).
[2] Huang C, Zhang H, Cao J, et al. Stability and Hopf bifurcation of a delayed prey–predator model with disease in the predator[J]. International Journal of Bifurcation and Chaos, 2019, 29(07): 1950091.
[3] Jie Cao, Fenghua Wen, H E Stanley, Xiong Wang. Multilayer financial networks and systemic importance: Evidence from China[J]. *International Review of Financial Analysis*, 2021: 101882.
